# Acute parvovirus B19-associated inflammatory musculoskeletal disease: clinical phenotypes and remission trajectories from the GIRRCS network

**DOI:** 10.1093/rheumatology/keag456

**Published:** 2026-08-21

**Authors:** Piero Ruscitti, Luisa Costa, Antonella Mastrangelo, Federica Viapiano, Marco Tasso, Giulia Sgarlata, Mario Cascone, Azadeh Shariat Panahi, Federico Perosa, Paola Cipriani, Giuliana Guggino, Roberto Giacomelli, Francesco Caso

**Affiliations:** Department of Biotechnological and Applied Clinical Sciences, University of L’Aquila, L’Aquila, Italy; Rheumatology Research Section, Department of Clinical Medicine and Surgery, University of Naples Federico II, Naples, Italy; Rheumatic and Systemic Autoimmune Diseases Unit, Department of Interdisciplinary Medicine, University of Bari Medical School, Bari, Italy; Laboratory of Cellular and Molecular Immunology, Department of Interdisciplinary Medicine, University of Bari Medical School, Bari, Italy; Department of Biotechnological and Applied Clinical Sciences, University of L’Aquila, L’Aquila, Italy; Rheumatology Research Section, Department of Clinical Medicine and Surgery, University of Naples Federico II, Naples, Italy; Department of Biotechnological and Applied Clinical Sciences, University of L’Aquila, L’Aquila, Italy; Rheumatology Research Section, Department of Clinical Medicine and Surgery, University of Naples Federico II, Naples, Italy; Department of Biotechnological and Applied Clinical Sciences, University of L’Aquila, L’Aquila, Italy; Rheumatic and Systemic Autoimmune Diseases Unit, Department of Interdisciplinary Medicine, University of Bari Medical School, Bari, Italy; Laboratory of Cellular and Molecular Immunology, Department of Interdisciplinary Medicine, University of Bari Medical School, Bari, Italy; Department of Biotechnological and Applied Clinical Sciences, University of L’Aquila, L’Aquila, Italy; Department of Health Promotion, Mother and Child Care, Internal Medicine and Medical Specialties, Rheumatology Section “P. Giaccone”, University of Palermo, Palermo, Italy; Clinical and research section of Rheumatology and Clinical Immunology, Fondazione Policlinico Universitario Campus Bio-Medico, Rome, Italy; Rheumatology and Clinical Immunology, Department of Medicine, University of Rome “Campus Biomedico”, School of Medicine, Rome, Italy; Rheumatology Research Section, Department of Clinical Medicine and Surgery, University of Naples Federico II, Naples, Italy; Immunopharmalab, Department of Pharmacy, School of Medicine and Surgery, University of Naples Federico II, Naples, Italy

**Keywords:** parvovirus B19, viral arthritis, acute arthritis, enthesitis, inflammatory musculoskeletal disease

## Abstract

**Objective:**

To describe clinical phenotype, selected imaging findings and remission trajectories in adults with acute parvovirus B19-associated inflammatory musculoskeletal disease referred for rheumatological assessment.

**Methods:**

This was a non-interventional multicentre observational study in GIRRCS rheumatology units. Adults with new-onset inflammatory musculoskeletal manifestations, positive anti-parvovirus B19 IgM and symptom onset within 4 weeks were enrolled. Clinical, laboratory and clinically indicated imaging data were collected. Time to first documented clinical remission was defined as the interval from symptom onset to the first rheumatologist-confirmed assessment showing resolution of inflammatory musculoskeletal manifestations. Patients requiring disease-modifying escalation were analysed separately.

**Results:**

Twenty-four adults were enrolled; 18 were women (75.0%) and mean age was 44.4 ± 14.8 years. Clinical involvement was polyarticular in 14 patients (58.3%) and oligoarticular in 10 (41.7%); enthesitis occurred in 4 (16.7%) and dactylitis was absent. Inflammatory markers were modestly elevated, and most patients with complete autoantibody data were seronegative. Imaging was performed selectively and documented inflammatory abnormalities in clinically indicated examinations. Follow-up data were available for 21 patients, of whom 20 (95.2%) achieved clinical remission without DMARD escalation; median documented remission timing was 21 days (IQR 10–60; range 7–135). One patient required biologic escalation for persistent inflammatory disease. Exploratory regression analyses did not identify robust associations with documented remission timing.

**Conclusion:**

Acute parvovirus B19-associated inflammatory musculoskeletal disease may mimic inflammatory rheumatic disease in adults referred for rheumatological assessment. Most patients achieved remission without disease-modifying escalation, although remission timing varied and one patient required biologic therapy for persistent inflammatory disease.

Rheumatology key messages

- Peripheral arthritis predominated; enthesitis and axial involvement were uncommon, selected observations.
- Imaging was clinically indicated and illustrative, not prevalence-defining.
- Remission was common without DMARD escalation, but documented timing was heterogeneous.

## Introduction

Human parvovirus B19 is a non-enveloped, single-stranded DNA virus transmitted predominantly through respiratory spread. After a period of relative epidemiological quiescence, parvovirus B19 has re-emerged as a pathogen of renewed clinical relevance, with surveillance data from Europe and the United States documenting a substantial increase in recent infections, particularly among children [1, 2]. Infection typically peaks during the first half of the year and is classically associated with erythema infectiosum in children, whereas its clinical relevance in adults remains underrecognized.

In immunocompetent adults, acute parvovirus B19 infection is often mild or asymptomatic, but it may also give rise to diverse systemic manifestations. More severe complications, including anaemia, neurological involvement and chronic cytopenias, occur predominantly in patients with haematological disorders or impaired immunity [2]. Of relevance to rheumatologists, parvovirus B19 may provoke musculoskeletal manifestations ranging from transient arthralgia to overt inflammatory arthritis and autoimmune-like phenotypes [3–5].

Published reports have described clinical patterns resembling rheumatoid arthritis, systemic lupus erythematosus, cryoglobulinaemic vasculitis and seronegative spondyloarthritis [6–9]. A recent study highlighted the risk of misclassifying acute parvovirus B19 infection as chronic systemic rheumatic disease, with the potential consequence of unnecessary immunosuppressive treatment [10]. These infectious mimics are increasingly recognized as a relevant diagnostic challenge in contemporary rheumatology [11, 12].

Recent multicentre data from Italy have highlighted the broad rheumatological heterogeneity associated with acute parvovirus B19 infection during the post-pandemic resurgence of the virus [13]. However, focused adult data describing the inflammatory musculoskeletal phenotype, rheumatologist-assessed clinical remission trajectories, and imaging findings in patients referred for rheumatological evaluation remain limited.

The present study was, therefore, designed to extend prior broader observations by providing focused musculoskeletal phenotyping of acute parvovirus B19-associated inflammatory musculoskeletal disease in adults referred for rheumatological assessment. Specifically, we aimed to describe clinical and laboratory features, time to first documented clinical remission using a predefined rheumatologist-assessed end point, treatment exposure and selected imaging findings from clinically indicated ultrasound and MRI examinations.

## Methods

### Study design and participants

This was a non-interventional multicentre observational study conducted over a 12-month period at rheumatology units participating in the GIRRCS network. Adults aged 18 years or older were enrolled if they presented with new-onset inflammatory musculoskeletal manifestations, including peripheral arthritis, enthesitis or inflammatory axial symptoms, in the setting of IgM-positive acute parvovirus B19 infection and symptom onset within 4 weeks before enrolment.

Acute parvovirus B19 infection was defined by positive anti-parvovirus B19 IgM serology in the context of a compatible recent clinical presentation and epidemiological or clinical circumstances considered consistent with recent infection. Serological testing was performed as part of routine clinical care at participating centres, during the initial diagnostic evaluation preceding or coinciding with enrolment. The diagnostic approach was based primarily on anti-parvovirus B19 IgM positivity in conjunction with clinical compatibility. Additional virological investigations, including parvovirus B19 PCR, were performed at the discretion of the treating physician when diagnostic uncertainty remained after clinical and serological assessment. Paired serology, IgG seroconversion, and avidity testing were not systematically required by the study protocol. To minimize the likelihood of persistent or false-positive IgM results, inclusion was restricted to patients with recent-onset inflammatory musculoskeletal manifestations and a clinical context considered compatible with acute parvovirus B19 infection by the treating physician.

Patients were excluded if they had a previous diagnosis of rheumatic disease, including autoimmune rheumatic disease, osteoarthritis or fibromyalgia, evidence of another active or clinically relevant latent infection, or known malignancy. The study protocol was approved by the Ethics Committee of the University of L’Aquila (Comitato Etico Azienda Sanitaria Locale 1 Avezzano/Sulmona/l’Aquila, L’Aquila, Italy; protocol no. 0095184/20), and the study was conducted in accordance with the Declaration of Helsinki. Written informed consent was obtained from all participants.

### Clinical, laboratory and imaging assessment

At baseline, demographic and clinical data were recorded, including age, sex, body-mass index, smoking status, comorbid conditions and any clinically significant infection other than parvovirus B19 occurring within the 4 weeks preceding symptom onset.

Musculoskeletal assessment included duration of musculoskeletal symptoms before enrolment, morning stiffness, tender-joint count, swollen-joint count and classification of articular involvement as peripheral, axial or mixed. Articular pattern was further categorized as oligoarticular or polyarticular according to the number of clinically involved joints at presentation. Enthesitis and dactylitis were systematically assessed.

Laboratory testing included erythrocyte sedimentation rate, C-reactive protein, antinuclear antibodies, anti-double-stranded DNA antibodies, extractable nuclear antigen antibodies, antiphospholipid antibodies, anti-neutrophil cytoplasmic antibodies, rheumatoid factor, anti-citrullinated peptide antibodies, complement levels and routine haematological and biochemical variables.

Imaging studies were performed according to clinical presentation and clinical indication and were not mandated systematically across the cohort. Ultrasound of symptomatic joints and entheses was performed using standardized scanning protocols. Synovitis was defined according to EULAR–OMERACT semiquantitative criteria as hypoechoic synovial hypertrophy, with or without effusion and graded Doppler signal [14]. Tenosynovitis was defined as anechoic or hypoechoic distension of the tendon sheath due to fluid or synovial hypertrophy, with or without Doppler signal. Erosions were recorded only when exceeding 2 mm. Enthesitis was defined according to the GRAPPA DUET sonographic framework [15].

Magnetic resonance imaging was performed according to clinical indication. Patients with axial symptoms underwent MRI of the sacroiliac joints and, when clinically indicated, the lumbar spine, using T1-weighted and fat-suppressed sequences, including short tau inversion recovery or T2-weighted fat-suppressed turbo spin-echo sequences, in accordance with ESSR recommendations [16]. Scans were interpreted according to ASAS/OMERACT criteria; active sacroiliitis was defined by subchondral bone marrow oedema and related inflammatory lesions, whereas structural lesions were defined by sclerosis and erosions [17].

Three patients with axial symptoms and MRI-confirmed sacroiliac involvement included in this cohort have been described previously in a separate case-based publication focused specifically on parvovirus B19-associated sacroiliitis [18]. They were retained in the present study because they were enrolled through the same GIRRCS network, fulfilled the same eligibility criteria, and formed part of the predefined multicentre cohort. In the present manuscript, these patients are included only in aggregate cohort-level analyses and to document selected imaging findings; detailed case-level descriptions of sacroiliac involvement have been reported previously [18].

### Follow-up and outcome

Participants underwent scheduled clinical reassessments at 3 and 6 months after enrolment. In addition, all documented clinically indicated outpatient assessments occurring before, between or after the scheduled visits during the 6-month observation period were reviewed to identify the first documented assessment at which complete clinical remission was confirmed.

Clinical remission was defined as complete resolution of clinically detectable inflammatory musculoskeletal manifestations, including articular and entheseal inflammatory signs and axial inflammatory manifestations when present, according to the treating rheumatologist’s assessment. Patient-reported symptoms alone were not used to define clinical remission.

Time to first documented clinical remission was calculated as the interval between symptom onset and the first documented clinical assessment at which complete clinical remission was confirmed during follow-up. Because remission timing was based on documented clinical assessments rather than continuous observation, this end point represents the timing of first clinically documented remission and not necessarily the exact biological time at which remission occurred.

Among patients in whom clinical remission was documented, remission occurring <30 days from symptom onset was classified descriptively as rapid first documented clinical remission, whereas remission occurring ≥30 days from symptom onset was classified as delayed first documented clinical remission. The <30-day threshold was selected pragmatically as an approximate 1-month descriptive landmark and was not intended as a validated prognostic cutoff. Patients requiring disease-modifying escalation because of persistent inflammatory disease were described separately.

Three patients were lost to follow-up and were excluded from longitudinal outcome analyses but retained in baseline descriptive analyses.

Patients who achieved clinical remission without escalation to conventional synthetic, biologic or targeted synthetic DMARD therapy were analysed as the remission-without-DMARD group. The patient who developed a persistent axial inflammatory disease course and subsequently initiated adalimumab after the 3-month reassessment was described separately as a biologic-escalation case. Because this patient did not achieve clinical remission under symptomatic treatment alone, the patient was not included in analyses restricted to remission without disease-modifying escalation, including remission-timing summaries for the remission-without-DMARD group and exploratory regression analyses.

### Statistical analysis

Continuous variables were summarized as means with standard deviations or medians with interquartile ranges, as appropriate. Categorical variables were reported as counts and percentages. Available-case denominators were used for variables with missing data.

Longitudinal outcomes were summarized descriptively among patients with available follow-up data according to documented remission timing and treatment escalation. First documented clinical remission occurring <30 days from symptom onset was classified descriptively as rapid remission, whereas remission documented ≥30 days from symptom onset without disease-modifying escalation was classified as delayed remission without DMARD escalation. The <30-day cutoff was selected pragmatically as an approximate 1-month descriptive landmark and was not intended as a validated prognostic threshold. The patient requiring biologic escalation for persistent axial inflammatory disease was analysed separately as a biologic-escalation case.

Remission-timing summaries and exploratory regression analyses were restricted to patients who achieved clinical remission without escalation to conventional synthetic, biologic, or targeted synthetic DMARD therapy. Candidate variables in univariable exploratory models included age, sex, smoking status, tender-joint count, swollen-joint count, erythrocyte sedimentation rate, C-reactive protein level and enthesitis. A parsimonious multivariable model included sex, swollen-joint count and C-reactive protein level.

Given the small sample size, right-skewed distribution of documented remission timing, multiple exploratory comparisons and non-continuous observation of remission, regression estimates were interpreted descriptively only and regarded as hypothesis-generating. No adjustment for multiple comparisons was applied. Regression analyses were not intended to identify independent predictors or determinants of documented remission timing.

Treatment exposure was summarized descriptively. Treatment variables were not included in regression models because NSAID use was universal among patients analysed for remission timing, no disease-modifying therapy was administered before remission in this subgroup, and the sample size was limited.

## Results

### Patient characteristics

Twenty-four adults with IgM-positive acute parvovirus B19 infection and new-onset inflammatory musculoskeletal manifestations were enrolled. Baseline, laboratory, imaging, treatment and exploratory outcome data are summarized in Table 1. Eighteen patients were women (75.0%). Mean age was 44.4 ± 14.8 years, with a median age of 44.5 years (IQR 36.8–49.2). Body-mass index, available for 23 patients, averaged 24.4 ± 4.2 kg/m². Fourteen patients (58.3%) reported no chronic comorbid conditions, and six patients (25.0%) were active smokers. No patient reported a clinically significant infection other than parvovirus B19 within the 4 weeks preceding symptom onset.

**Table 1:** Baseline, laboratory, imaging, treatment and exploratory outcome data from the multicentre GIRRCS study of acute parvovirus B19-associated inflammatory musculoskeletal disease.

Variable	Value
Baseline cohort (*N* = 24)	
Family history of autoimmune or rheumatic disease, no./total no. (%)^a^	1/10 (10.0)
Thyroid nodular disease, no. (%)	6 (25.0)
Osteoporosis, no. (%)	2 (8.3)
Hypovitaminosis D, no. (%)	2 (8.3)
Pulmonary or thromboembolic disorders, any, no. (%)	2 (8.3)
Asthma, no. (%)	1 (4.2)
History of pulmonary embolism, no. (%)	1 (4.2)
Arterial hypertension, no. (%)	3 (12.5)
No clinically significant infection other than parvovirus B19 within the 4 weeks preceding symptom onset, no. (%)	24 (100.0)
ESR, median (IQR), mm/h	45 (21–56)
CRP, median (IQR), mg/dl	2.65 (1.75–3.25)
Ultrasound: normal findings, no./total no. (%)^b^	1/7 (14.3)
Patients with available follow-up outcome data (*N* = 21)	
Clinical remission without DMARD escalation, no. (%)	20 (95.2)
Rapid first documented clinical remission <30 days, no. (%)	9 (42.9)
Delayed first documented clinical remission without DMARD escalation ≥30 days, no. (%)	11 (52.4)
Persistent inflammatory disease requiring biologic escalation, no. (%)	1 (4.8)
No systemic glucocorticoid use during follow-up, no. (%)	21 (100.0)
Remission-without-DMARD group (*N* = 20)	
Documented remission timing, mean ± SD, days	37.9 ± 35.6
Documented remission timing, median (IQR), days	21 (10–60)
Documented remission timing, range, days	7–135
Rapid first documented clinical remission <30 days, no. (%)	9 (45.0)
Delayed first documented clinical remission ≥30 days, no. (%)	11 (55.0)
No conventional synthetic, biologic, or targeted synthetic DMARD before remission, no. (%)	20 (100.0)
Exploratory regression estimates in the remission-without-DMARD group (*N* = 20)^c^	
Male sex vs. female sex, univariable *β*, days	−30.0
Swollen-joint count, univariable *β*, days per joint	+1.63
CRP, univariable *β*, days per 1 mg/l	+2.86
CRP, adjusted *β*, days per 1 mg/l^d^	+2.95

CRP: C-reactive protein; DMARD: disease-modifying antirheumatic drug; ESR: erythrocyte sedimentation rate; IQR: interquartile range; SD: standard deviation.

a Family history data were available for 10 patients.

b Ultrasound was performed only when clinically indicated and was not mandated systematically across the cohort.

c Exploratory regression analyses were restricted to patients who achieved clinical remission without escalation to conventional synthetic, biologic or targeted synthetic DMARD therapy. The dependent variable was documented remission timing, defined as the interval between symptom onset and the first documented clinical assessment at which complete clinical remission was confirmed. Positive *β* coefficients indicate longer documented remission timing; negative *β* coefficients indicate shorter documented remission timing. These analyses were exploratory and interpreted descriptively only; they were not intended to identify independent predictors or determinants of documented remission timing. The <30-day threshold was selected pragmatically as an approximate 1-month descriptive landmark and was not intended as a validated prognostic cutoff.

d From the exploratory multivariable model including sex, swollen-joint count and CRP.

### Clinical and laboratory findings

Mean tender-joint count was 9.9 ± 9.6, with a median of 7 (IQR 2–15), whereas mean swollen-joint count was 4.9 ± 8.3, with a median of 1 (IQR 0–5). Oligoarticular involvement was present in 10 patients (41.7%), and polyarticular involvement in 14 patients (58.3%). Enthesitis was observed in 4 of 24 patients (16.7%) and axial symptoms were reported in 3 patients (12.5%). Dactylitis was not observed.

Mean erythrocyte sedimentation rate was 40.6 ± 21.9 mm/h, and mean C-reactive protein was 3.96 ± 4.41 mg/dl. Among the 21 patients with complete autoantibody data, 19 were seronegative for the assessed autoantibody panel (90.5%) and 2 showed isolated low-titre ANA positivity at 1:80 (9.5%), without additional autoantibody abnormalities. Autoantibody data were incomplete or unavailable in 3 of 24 patients (12.5%).

### Imaging findings

Imaging was performed selectively according to clinical presentation and clinical indication and was not intended to define the prevalence or full imaging spectrum of parvovirus B19-associated inflammatory musculoskeletal disease. Ultrasound was performed in 7 of 24 patients (29.2%) and documented synovitis or effusion in 2 patients, tenosynovitis in 4 patients, and enthesitis in 4 patients; these findings were not mutually exclusive, and 1 patient had normal ultrasound findings.

Magnetic resonance imaging was performed in 7 of 24 patients (29.2%) according to clinical indication, including sacroiliac MRI in the 3 patients with axial symptoms and peripheral MRI in 4 patients. Active sacroiliitis was identified in all 3 patients evaluated for axial symptoms, and structural erosive changes were observed in one of them.

### Clinical course and treatment exposure

Follow-up outcome data were available for 21 of the 24 enrolled patients; 3 patients were lost to follow-up. During follow-up, 20 of 21 patients with available outcome data achieved clinical remission without escalation to conventional synthetic, biologic, or targeted synthetic DMARD therapy (95.2%). The remaining patient had persistent MRI-confirmed erosive sacroiliitis, did not achieve clinical remission with symptomatic treatment, and developed a persistent SpA-like axial inflammatory course. Adalimumab was initiated 168 days after symptom onset and was followed by clinical improvement. As previously reported, subsequent MRI performed after biologic initiation showed complete resolution of bone marrow oedema [18].

Using the descriptive threshold of <30 days, 9 of 21 patients with available follow-up data achieved rapid first documented clinical remission (42.9%). Of the remaining 12 patients, 11 achieved delayed first documented clinical remission without disease-modifying escalation (≥30 days; 52.4%), and 1 had persistent inflammatory disease requiring biologic escalation after the 3-month reassessment.

Among the 20 patients who achieved clinical remission without disease-modifying escalation, documented remission timing ranged from 7 to 135 days, with a mean of 37.9 ± 35.6 days and a median of 21 days (IQR 10–60). In this remission-without-DMARD group, 9 patients achieved remission <30 days from symptom onset (45.0%) and 11 achieved remission ≥30 days (55.0%). This group was used for remission-timing summaries and exploratory regression analyses. Selected exploratory regression estimates are summarized in Table 1.

All enrolled patients received non-steroidal anti-inflammatory drugs during the acute phase according to routine clinical practice. No patient with available follow-up data received systemic glucocorticoids during follow-up, and no patient in the remission-without-DMARD group received conventional synthetic, biologic or targeted synthetic DMARD therapy before remission. Thus, variability in documented remission timing occurred within an NSAID-based symptomatic treatment strategy rather than in an untreated cohort.

**Figure 1:**
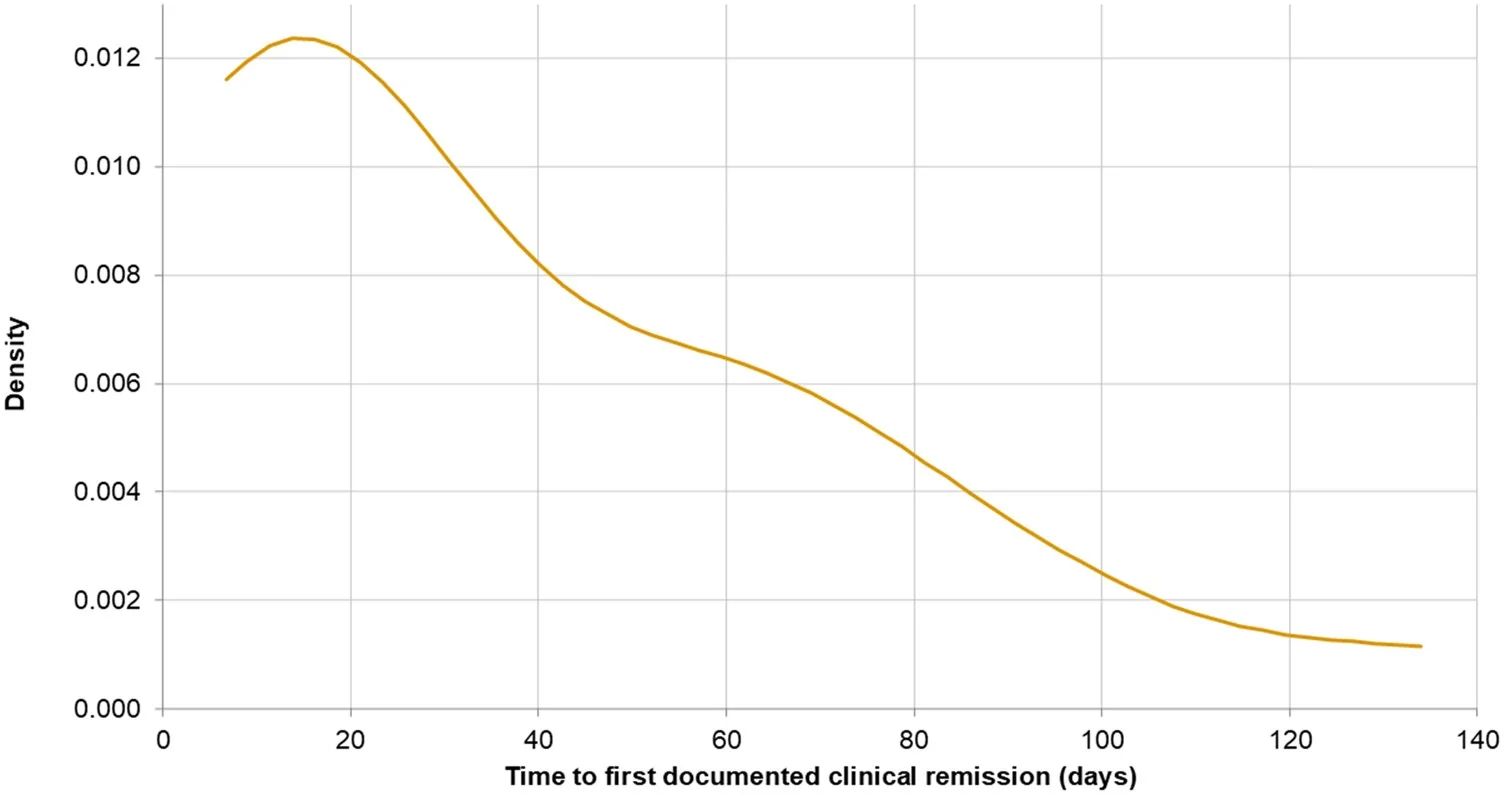
Distribution of time to first documented clinical remission. Kernel-density plot showing the smoothed overall distribution of time to first documented clinical remission among the 20 patients who achieved clinical remission during follow-up without escalation to conventional synthetic, biologic, or targeted synthetic disease-modifying antirheumatic drug therapy. The curve illustrates a heterogeneous distribution of documented remission timing, with greater density at earlier time points and a right-sided tail extending to 135 days. Kernel-density smoothing was used only for descriptive visualization and was not used to infer subgroup patterns, predictors, determinants, or stable distributional features

The distribution of documented remission timing in the remission-without-DMARD group was heterogeneous, with greater density at earlier time points and a right-sided tail extending to 135 days (Fig. 1).

### Exploratory regression analyses

Exploratory regression analyses were conducted among the 20 patients who achieved clinical remission during follow-up without escalation to conventional synthetic, biologic or targeted synthetic DMARD therapy and who therefore had observed documented-remission timing before any disease-modifying escalation. No clear association with documented remission timing was identified in these exploratory analyses. Baseline C-reactive protein, swollen-joint count, and male sex showed descriptive point estimates for documented remission timing; however, given the very small sample size, right-skewed outcome distribution, multiple exploratory comparisons and exploratory nature of these analyses, these findings should be interpreted cautiously and regarded as hypothesis-generating only. Selected exploratory regression estimates are summarized in Table 1.

## Discussion

In this multicentre observational study, acute parvovirus B19-associated inflammatory musculoskeletal disease in adults referred for rheumatological evaluation was characterized predominantly by peripheral arthritis [3–5, 11]. Tenosynovitis, enthesitis and axial involvement were documented only in selected clinically indicated assessments. The three patients with MRI-confirmed sacroiliac involvement had been reported previously [18] and were retained because they belonged to the same GIRRCS cohort, fulfilled the same eligibility criteria and are presented here only in aggregate analyses.

This referral-based study was designed to describe focused musculoskeletal phenotypes, selected imaging findings and rheumatologist-assessed remission trajectories, rather than to estimate population-level frequencies in all adults with acute parvovirus B19 infection. Consistently, because ultrasound and MRI were performed selectively according to clinical indication, imaging findings should be interpreted as illustrative observations rather than as systematic imaging characterization. In particular, the small number of axial cases precludes conclusions regarding the frequency, prevalence or broader clinical significance of sacroiliac involvement in acute parvovirus B19 infection.

The main clinical relevance of these findings lies in the overlap with early chronic inflammatory rheumatic disease, including seronegative spondyloarthritis, undifferentiated inflammatory arthritis and systemic rheumatic disease [6–12]. Recognition of the temporal association with acute parvovirus B19 infection may help avoid premature diagnostic classification and unnecessary long-term immunomodulatory treatment [10–12].

The clinical course was favourable in most patients, although documented remission timing was heterogeneous. Most patients achieved clinical remission without disease-modifying escalation, with documented remission timing ranging from 7 to 135 days in the remission-without-DMARD group. One patient required biologic therapy for persistent inflammatory disease, as previously reported [18], and was analysed separately from this group.

Treatment exposure is relevant to the interpretation of documented remission timing. All patients received NSAIDs during the acute phase; no patient with follow-up data received systemic glucocorticoids, and no patient in the remission-without-DMARD group received conventional synthetic, biologic or targeted synthetic DMARD therapy before remission. Therefore, documented remission timing should be interpreted within an NSAID-based symptomatic treatment context, rather than as the untreated natural history of the disease.

Exploratory regression analyses did not identify robust associations with documented remission timing. In view of the limited sample size, right-skewed outcome distribution, multiple exploratory comparisons and restriction to patients achieving remission without disease-modifying escalation, the observed estimates for baseline C-reactive protein, swollen-joint count and male sex should be interpreted descriptively and regarded as hypothesis-generating rather than prognostic.

Strengths include the multicentre design, structured clinical and laboratory characterization, focus on an under-recognized infectious mimic, selected illustrative imaging observations and use of a rheumatologist-assessed remission end point. Limitations include the modest sample size, three patients lost to follow-up, non-continuous observation of remission, IgM-based diagnosis without systematic PCR, paired serology, IgG seroconversion or avidity testing, selective and locally interpreted imaging, referral-based recruitment, limited treatment-exposure detail and the very small number of axial cases. These factors limit precision, causal interpretation, prevalence estimation and generalizability.

In conclusion, this rheumatology-referred adult cohort describes recent-onset inflammatory musculoskeletal manifestations associated with IgM-positive acute parvovirus B19 infection. The clinical presentation overlapped with features of early chronic inflammatory rheumatic disease. Clinical remission without disease-modifying escalation was documented in most patients with available follow-up, although remission timing was heterogeneous and one patient required biologic therapy for persistent inflammatory disease. These observations should be interpreted in light of the referral-based design, selective imaging, limited sample size and IgM-based diagnostic approach.

## Data Availability

The data underlying this article will be shared on reasonable request to the corresponding author.
